# Population-Based Analysis of Demographic and Socioeconomic Disparities in Pediatric CNS Cancer Survival in the United States

**DOI:** 10.1038/s41598-020-61237-2

**Published:** 2020-03-12

**Authors:** Robert Fineberg, Shadi Zahedi, Megan Eguchi, Muriel Hart, Myles Cockburn, Adam L. Green

**Affiliations:** 1 University of Colorado School of Medicine, Aurora, CO USA; 2 Morgan Adams Foundation Pediatric Brain Tumor Research Program, Aurora, CO USA; 3 University of Colorado Comprehensive Cancer Center, Aurora, CO USA; 4 Center for Cancer and Blood Disorders, Children’s Hospital Colorado, Aurora, CO USA

**Keywords:** Paediatric cancer, Epidemiology

## Abstract

Previous studies have demonstrated effects of racial and socioeconomic factors on survival of adults with cancer. While less studied in the pediatric population, data exist demonstrating disparities of care and survival in pediatric oncology patients based on socioeconomic and racial/ethnic factors. Brain cancers recently overtook leukemia as the number one cause of childhood cancer fatalities, but demographic and socioeconomic disparities in these tumors have not been adequately studied. We obtained data from the SEER Program of the National Cancer Institute (NCI). We selected patients under 19 years of age with central nervous system (CNS) cancers diagnosed between 2000 and 2015. We included patient demographics, tumor characteristics, treatment, and socioeconomic characteristics as covariates in the analysis. We measured overall survival and extent of disease at diagnosis. We saw that Black and Hispanic patients overall had a higher risk of death than non-Hispanic White patients on multivariable analysis. On stratified analysis, Black and Hispanic patients with both metastatic and localized disease at diagnosis had a higher risk of death compared to White, non-Hispanic patients, although the difference in Black patients was not significant after adjusting for mediating factors. However, our findings on extent of disease at diagnosis demonstrated that neither Black race nor Hispanic ethnicity increased the chance of metastatic disease at presentation when controlling for mediating variables. In summary, racial and ethnic disparities in childhood CNS tumor survival appear to have their roots at least partially in post-diagnosis factors, potentially due to the lack of access to high quality care, leading to poorer overall outcomes.

## Introduction

Previous studies have demonstrated effects of racial and socioeconomic factors on survival of adults with cancer^1–3^. For instance, it has been shown that access to private insurance compared to Medicaid or no insurance positively affects the prognosis of adult glioblastoma patients leading to better overall survival^4^. While less studied in the pediatric population, data exist demonstrating disparities of care and survival in pediatric oncology patients based on socioeconomic and racial/ethnic factors^5–7^. In the U.S., malignant neoplasms are the leading cause of death by disease in children past infancy. Brain cancers recently overtook leukemia as the number one cause of childhood cancer fatalities, despite being less common^8^. This is due to improvements in leukemia treatment and reduction of mortality in these patients, as well as the relative stagnation of improved outcomes in brain cancer patients. Despite improvement of overall survival in leukemia patients, children of lower socioeconomic status have benefitted less than their higher income contemporaries^9^. Length of time to diagnosis and treatment modalities influence survival of children with various cancers^10,11^. Additionally, treatment factors such as extent of tumor resection, tumor location, age, and year of diagnosis have been shown to be associated with survival in pediatric glioblastoma patients^12^. However, little attention has been devoted to specific demographic and socioeconomic risk factors contributing to survival of childhood brain cancers as a group, and how these factors may influence survival. In this context, we hypothesized that demographic and socioeconomic disparities may impair access to care and/or quality of care in childhood brain cancers, leading to poorer survival. In this study, we examined the effect of demographic and socioeconomic factors on survival in pediatric brain tumors using the Surveillance, Epidemiology, and End Results (SEER) database. We analyzed overall survival and stage at diagnosis for subjects 0–19 years of age, based on race, ethnicity, socioeconomic status, and other demographic variables to determine risk factors and mechanisms of poorer outcomes in this group.

## Methods

### Data collection

Data were obtained from the SEER Program of the National Cancer Institute (NCI). SEER collects information from population-based cancer registries that currently cover approximately 28 percent of the U.S. population. Available data include information on patient demographics, tumor characteristics at diagnosis, treatment, survival time based on linkage to mortality data from the National Center for Health Statistics, and county-level socioeconomic information based on census data.

We selected patients between the ages of 0 and 19 years with central nervous system (CNS) tumors (International Classification of Diseases for Oncology 3^rd^ edition (ICD-O-3)^13^ topography codes C70.0-C70.9, C71.0-C71.9, C72.0-C72.9, C75.1, and C75.3) diagnosed between 2000 and 2015 from the SEER 18 Regs Custom Data, Nov 2017 Submission using SEER*Stat Version 8.3.5. We selected only those tumors with malignant behavior and only the first occurrence of cancer. We limited the following International Classification of Childhood Cancer, Third edition (ICCC-3)^14^ site recodes to Grades III and IV: III(b) Astrocytomas, III(d.1) Oligodendrogliomas, III(d.2) Mixed and unspecified gliomas, III(a.2) Choroid plexus tumor, III(d.3) Neuroepithelial glial tumors of uncertain origin, III(e.4) Neuronal and mixed neuronal-glial tumors, III(e.5) Meningiomas, III(f) Unspecified intracranial and intraspinal neoplasms. For the remaining ICCC-3 CNS site recodes, all patients with known grade were included.

We excluded cases reported on death certificates or autopsies only, cases with zero days of survival, and cases for whom this was not the patient’s first primary malignant tumor. The remaining cases were categorized according to the ICCC-3 site recode into five tumor types: (1) ependymomas, (2) gliomas (including astrocytomas, oligodendrogliomas, and mixed and unspecified gliomas), (3) PNET/Pineal/ATRT (including primitive neuroectodermal tumors (PNET), atypical teratoid/rhabdoid tumors (ATRT), and pineal parenchymal tumors), and (4) medulloblastomas (including medulloblastomas and medulloepithelioma). Other/unspecified tumors (including chorioid plexus tumors, germ cell tumors, neuroepithelial glial tumors of uncertain origin, neuronal and mixed neuronal-glial tumors, meningiomas, and unspecified intracranial and intraspinal neoplasms) were included in the overall analysis, but because of the heterogeneity of this group, these tumors were not included in subgroup analysis.

We included year of diagnosis, patient demographics, tumor characteristics, treatment, and socioeconomic characteristics as covariates in the analysis. Patient characteristics included year of diagnosis, sex (male and female), race (White, Black, and Other), Hispanic ethnicity, age group (0 years, 1 to 4 years, 5 to 9 years, 10 to 14 years, and 15 to 19 years), and insurance status (private versus public or no insurance; note that insurance information was only available beginning in 2007). Tumor characteristics included SEER Summary Stage (localized versus regional or distant, which together composed the metastatic category) and tumor size (less than 6 cm, 6 cm or more, and unknown). Treatment variables were limited to treatment at initial diagnosis and included the use of radiation therapy and surgery at the primary site. Chemotherapy was not included as a variable because it is not a standard measure available through SEER and, when collected, is not captured as sensitively as data on radiation therapy^15^.

County-level socioeconomic characteristics were obtained from 2007–2011 Census American Community Survey data and included percent population with less than a high school degree, percent of families below the poverty line, and percent of households in language isolation. The Census Bureau considers a household to be linguistically isolated when all members above the age of 14 speak a non-English language and speak English less than “very well.” For these socioeconomic characteristics, patients were categorized by whether the patient’s county was in the most disadvantaged quartile for the study population.

### Statistical analysis

We measured overall survival as the number of months from diagnosis to death due to any cause. Patients who were alive at the last follow-up were censored at the date of last follow-up, and patients surviving more than five years were censored at 60 months.

We performed statistical analyses using SAS software, Version 9.4 (SAS Institute Inc., Cary, NC), and significance was defined at a p-value <0.05. Chi-square tests were used to evaluate bivariate associations between covariates and tumor type. Multivariable logistic regression was used to evaluate the effects of demographic, socioeconomic, and tumor characteristics on metastatic disease at diagnosis and overall survival, as well as the use of radiation and surgery. Kaplan-Meier survival curves and the log-rank test were used to evaluate univariate effects on survival. Hazard ratios (HR) were obtained using Cox proportional hazards for univariate and multivariable survival analyses. The proportional hazards assumption was evaluated using Schoenfeld residuals and violations were addressed using time-dependent interaction terms in multivariable models.

## Results

A total of 1,881 unique records were selected from the SEER database with a diagnosis of malignant tumors of the CNS, including both cranial and spinal neoplasms. Patient characteristics, demographics, and disease categories are shown in Table 1. Of the total number of patients, Whites comprise 78.15% (1,470/1,881) of the cohort, Blacks 13.18%, non-Hispanics 72.09%, and Hispanics 27.91%. To study the effect of socioeconomic factors on survival, patients in the highest quartile of the sample for percent with high school or less education, percent below poverty level, and percent language isolation were considered the most disadvantaged. Gliomas were the most common tumors (n = 788), followed by ependymomas and medulloblastomas.

**Table 1:** Baseline data distribution.

Variable	Attribute	Overall	% with Attribute	Ependymoma	% with Attribute	Glioma	% with Attribute
		N		N		N	
All Cases		1881		418		788	
Sex	Female	846	44.98	204	48.80	359	45.56
	Male	1035	55.02	214	51.20	429	54.44
Race	White	1470	78.15	320	76.56	607	77.03
	Black	248	13.18	57	13.64	115	14.59
	Other	154	8.19	40	9.57	62	7.87
	Unknown	9	0.48	1	0.24	4	0.51
Ethnicity	Non-Hispanic	1356	72.09	292	69.86	576	73.10
	Hispanic	525	27.91	126	30.14	212	26.90
Age at Diagnosis	00 years	111	5.90	28	6.70	33	4.19
	01–04 years	496	26.37	166	39.71	110	13.96
	05–09 years	515	27.38	98	23.44	217	27.54
	10–14 years	372	19.78	68	16.27	186	23.60
	15–19 years	387	20.57	58	13.88	242	30.71
Tumor site	Ependymoma	418	22.22				
	Glioma	788	41.89				
	PNET/Pineal/ATRT	196	10.42				
	Medulloblastoma	393	20.89				
	Other/Unspecified	86	4.57				
Tumor size	Less than 6 cm	1072	56.99	225	53.83	406	51.52
	6 cm or greater	252	13.40	85	20.33	100	12.69
Extent of Disease at Diagnosis	Localized	1337	71.08	319	76.32	582	73.86
	Regional/Distant	459	24.40	90	21.53	189	23.98
	Unknown	85	4.52	9	2.15	17	2.16
	Missing	557	29.61	108	25.84	282	35.79
Radiation	No Radiation	546	29.03	116	27.75	227	28.81
	Radiation Administered	1317	70.02	299	71.53	554	70.30
	Unknown	18	0.96	3	0.72	7	0.89
Surgery	No Surgery	269	14.30	8	1.91	222	28.17
	Surgery	1610	85.59	409	97.85	566	71.83
	Unknown	2	0.11	1	0.24	0	0.00
Percent HS or less	All Other HS or less	1348	71.66	296	70.81	552	70.05
	Highest Quartile % HS or less	533	28.34	122	29.19	236	29.95
Percent Below Poverty Level	All Other Below Poverty Level	1318	70.07	287	68.66	540	68.53
	Highest Quartile % Below Poverty Level	563	29.93	131	31.34	248	31.47
Percent Language Isolation	All Other Language Isolation	1376	73.15	298	71.29	572	72.59
	Highest Quartile % Language Isolation	505	26.85	120	28.71	216	27.41
Insurance Status	Public/No Insurance	385	36.15	88	37.77	167	38.30
	Private Insurance	670	62.91	143	61.37	265	60.78
	Unknown	10	0.94	2	0.86	4	0.92
Variable	Attribute	PNET/Pineal/ATRT		Medulloblastoma		Unspecified/Other	
		N	% with Attribute	N	% with Attribute	N	% with Attribute
All Cases		196		393		86	
Sex	Female	94	47.96	160	40.71	29	33.72
	Male	102	52.04	233	59.29	57	66.28
Race	White	146	74.49	328	83.46	69	80.23
	Black	31	15.82	35	8.91	10	11.63
	Other	17	8.67	29	7.38	6	6.98
	Unknown	2	1.02	1	0.25	1	1.16
Ethnicity	Non-Hispanic	143	72.96	283	72.01	62	72.09
	Hispanic	53	27.04	110	27.99	24	27.91
Age at Diagnosis	00 years	26	13.27	15	3.82	9	10.47
	01–04 years	77	39.29	123	31.30	20	23.26
	05–09 years	41	20.92	142	36.13	17	19.77
	10–14 years	27	13.78	70	17.81	21	24.42
	15–19 years	25	12.76	43	10.94	19	22.09
Tumor size	Less than 6 cm	104	53.06	298	75.83	39	45.35
	6 cm or greater	32	16.33	16	4.07	19	22.09
Extent of Disease at Diagnosis	Localized	99	50.51	274	69.72	63	73.26
	Regional/Distant	54	27.55	113	28.75	13	15.12
	Unknown	43	21.94	6	1.53	10	11.63
	Missing	60	30.61	79	20.10	28	32.56
Radiation	No Radiation	72	36.73	88	22.39	43	50.00
	Radiation Administered	121	61.73	301	76.59	42	48.84
	Unknown	3	1.53	4	1.02	1	1.16
Surgery	No Surgery	24	12.24	7	1.78	8	9.30
	Surgery	172	87.76	385	97.96	78	90.70
	Unknown	0	0.00	1	0.25	0	0.00
Percent HS or less	All Other HS or less	138	70.41	296	75.32	66	76.74
	Highest Quartile % HS or less	58	29.59	97	24.68	20	23.26
Percent Below Poverty Level	All Other Below Poverty Level	133	67.86	298	75.83	60	69.77
	Highest Quartile % Below Poverty Level	63	32.14	95	24.17	26	30.23
Percent Language Isolation	All Other Language Isolation	141	71.94	302	76.84	63	73.26
	Highest Quartile % Language Isolation	55	28.06	91	23.16	23	26.74
Insurance Status	Public/No Insurance	35	33.65	83	34.30	12	24.00
	Private Insurance	69	66.35	156	64.46	37	74.00
	Unknown	0	0.00	3	1.24	1	2.00

HS = high school; N = number of individuals in each category.

Note: Highest Quartile % of county-level factors indicates the greatest level of disadvantage.

### Univariate analysis

We first undertook a univariate analysis to determine which factors correlated with risk of death (Table 2). Age, race, ethnicity, tumor type, extent of disease, and living in areas with higher poverty all had a significant impact on risk of death. Survival was worse for Black compared to White patients (HR: 1.31, 95% CI: 1.08–1.58, p-value = 0.0064) and worse for Hispanic compared to non-Hispanic patients (HR: 1.25, 95% CI: 1.07–1.45, p-value = 0.0038). Areas of high poverty exhibited worse overall survival (HR: 1.26, 95% CI: 1.09–1.46, p-value = 0.0019). The risk of death was also greater in subjects with metastatic disease compared to localized, and ages 0 and 5–9 years compared to age 1–4 years. The risk of death was lower in all tumor subtypes compared to gliomas and lower for subjects who underwent surgery compared to those who did not. Tumor size, the remaining socioeconomic measures, and radiation administration did not show significant association with overall survival.

**Table 2:** Overall survival in univariate and multivariable analysis.

Variable	Univariate analysis								
	Overall								
	N	5-yr Survival Estimate		Median Survival Months	All (Log-Rank p-value)		HR	HR 95% CI	p-value
Year of Diagnosis									
Female (ref) Male	846 1035	53.86% 49.28%		— 57	0.0819		1.13	(0.98, 1.30)	0.0846
White (ref) Black Other	1470 248 154	52.28% 44.18% 52.77%		— 34 —	**0.0197**		1.31 0.98	(1.08, 1.58) (0.75, 1.27)	**0.0064** 0.8548
Non-Hispanic (ref) Hispanic	1356 525	53.42% 45.56%		— 39	**0.0035**		1.25	(1.07, 1.45)	**0.0038**
00 years 01–04 years (ref) 05–09 years 10–14 years 15–19 years	111 496 515 372 387	48.87% 57.65% 46.60% 48.82% 52.53%		59 — 48 56 —	**0.0041**		1.55 1.34 1.21 1.06	(1.14, 2.10) (1.11, 1.62) (0.98, 1.49) (0.86, 1.30)	**0.0048** **0.0028** 0.0704 0.6138
Gliomas (ref) Ependymoma PNET/Pineal/ATRT Medulloblastoma Unspecified/other	788 418 196 393 86	32.48% 70.98% 51.21% 64.77% 71.28%		21 — — — —	**<0.0001**		0.27 0.63 0.37 0.32	(0.22, 0.34) (0.50, 0.80) (0.30, 0.46) (0.21, 0.49)	**<0.0001** **0.0001** **<0.0001** **<0.0001**
Less than 6 cm (ref) 6 cm or greater Missing	1072 252	54.33% 46.90%		— 49	0.0826		1.20	(0.98, 1.47)	0.0852
Localized (ref) Regional/Distant	1337 459	54.64% 39.03%		— 26	**<0.0001**		1.59	(1.37, 1.85)	**<0.0001**
No Radiation (ref) Radiation Administered	546 1317	55.87% 49.44%		— 58	0.7172		1.03	(0.88, 1.20)	0.7209
No Surgery (ref) Surgery	269 1610	22.44% 56.24%		13 —	**<0.0001**		0.34	(0.29, 0.40)	**<0.0001**
All Other HS or less (ref) Highest Quartile % HS or less	1348 533	53.12% 46.81%		— 51	0.0534		1.16	(1.00, 1.35)	0.0555
All Other Below Poverty Level (ref) Highest Quartile % Below Poverty Level	1318 563	53.83% 45.38%		— 41	**0.0017**		1.26	(1.09, 1.46)	**0.0019**
All Other Language Isolation (ref) Highest Quartile % Language Isolation	1376 505	52.76% 47.30%		— 52	0.1427		1.12	(0.96, 1.31)	0.1462
Private Insurance (ref) Public/No Insurance	670 385	55.70% 47.72%		— 53	0.0932		1.19	(0.97, 1.46)	0.0960
Variable	Multivariable analysis								
	Controlled for: Demographic and Tumor Characteristics (N = 1787)			Controlled for: Demographic, Tumor, and Treatment Characteristics (N = 1769)			Controlled for: Demographic, Tumor, Treatment, and SES Characteristics (N = 1769)		
	HR	HR 95% CI	p-value	HR	HR 95% CI	p-value	HR	HR 95% CI	p-value
Year of Diagnosis	0.99	(0.97, 1.01)	0.2321	0.99	(0.97, 1.00)	0.1021	0.99	(0.97, 1.00)	0.1409
Female (ref) Male	1.15	(1.00, 1.33)	0.0529	1.13	(0.98, 1.31)	0.0936	1.14	(0.99, 1.32)	0.0696
White (ref) Black Other	1.39 1.09	(1.14, 1.70) (0.83, 1.43)	**0.0014** 0.5329	1.36 1.09	(1.11, 1.67) (0.83, 1.43)	**0.0035** 0.5243	1.29 1.06	(1.04, 1.59) (0.80, 1.40)	**0.0206** 0.6765
Non-Hispanic (ref) Hispanic	1.36	(1.16, 1.60)	**0.0002**	1.33	(1.13, 1.57)	**0.0006**	1.29	(1.08, 1.53)	**0.0051**
00 years 01–04 years (ref) 05–09 years 10–14 years 15–19 years	1.66 0.95 0.67 0.37	(1.20, 2.29) (0.77, 1.17) (0.51, 0.87) (0.27, 0.53)	**0.0022** 0.6366 **0.0028** **<0.0001**	1.82 0.92 0.7 0.42	(1.31, 2.54) (0.74, 1.14) (0.53, 0.93) (0.30, 0.60)	**0.0004** 0.45 **0.0131** **<0.0001**	1.9 0.92 0.71 0.42	(1.36, 2.66) (0.74, 1.15) (0.54, 0.94) (0.30, 0.61)	**0.0002** 0.4746 **0.0173** **<0.0001**
Gliomas (ref) Ependymoma PNET/Pineal/ATRT Medulloblastoma Unspecified/other	0.22 0.7 0.4 0.63	(0.17, 0.28) (0.53, 0.91) (0.31, 0.51) (0.35, 1.14)	**<0.0001** **0.0069** **<0.0001** 0.1252	0.23 0.76 0.46 0.83	(0.17, 0.29) (0.58, 0.99) (0.35, 0.60) (0.46, 1.49)	**<0.0001** **0.044** **<0.0001** 0.5315	0.22 0.75 0.45 0.82	(0.17, 0.29) (0.58, 0.99) (0.34, 0.60) (0.46, 1.46)	**<0.0001** **0.0385** **<0.0001** 0.4946
Less than 6 cm (ref) 6 cm or greater Missing	1.13 1.07	(0.92, 1.40) (0.91, 1.26)	0.2541 0.4063	1.2 1.02	(0.96, 1.48) (0.86, 1.20)	0.1028 0.8474	1.19 1.02	(0.96, 1.47) (0.87, 1.20)	0.118 0.804
Localized (ref) Regional/Distant	1.61	(1.38, 1.88)	**<0.0001**	1.51	(1.29, 1.76)	**<0.0001**	1.51	(1.29, 1.77)	**<0.0001**
No Radiation (ref) Radiation Administered				0.56	(0.42, 0.74)	**<0.0001**	0.55	(0.41, 0.74)	**<0.0001**
No Surgery (ref) Surgery				0.39	(0.29, 0.51)	**<0.0001**	0.39	(0.29, 0.52)	**<0.0001**
All Other HS or less (ref) Highest Quartile % HS or less							0.92	(0.74, 1.15)	0.4674
All Other Below Poverty Level (ref) Highest Quartile % Below Poverty Level							1.27	(1.03, 1.57)	**0.0282**
All Other Language Isolation (ref) Highest Quartile % Language Isolation							0.79	(0.60, 1.04)	0.0967
Time-dependent Site Time-dependent Age Time-dependent Radiation Time-dependent Surgery Time-dependent Highest Quartile Language Isolation	0.99 1.01	(0.99, 1.00) (1.01, 1.02)	**0.0125** **<0.0001**	0.99 1.01 1.05 1.02	(0.99, 1.00) (1.00, 1.01) (1.04, 1.07) (1.00, 1.04)	**0.0020** **0.0001** **<0.0001** **0.0144**	0.99 1.01 1.06 1.02 1.02	(0.99, 1.00) (1.00, 1.01) (1.04, 1.07) (1.00, 1.04) (1.00, 1.03)	**0.0019** **0.0002** **<0.0001** **0.0116** **0.0053**

CI = confidence interval; HR = hazard ratio; HS = high school; N = number of individuals in each category.

Notes: Highest quartile % of county-level factors indicates the greatest level of disadvantage. Hazard ratio (HR) indicates the ratio of hazard of death by levels of covariates. Significant values (p < 0.05) have been marked with bold.

In tumor subtype analyses (Supplemental Table 1), significant effects included a greater risk for death in ependymoma and medulloblastoma patients with non-private insurance compared to privately insured subjects, and in Hispanic compared to non-Hispanic patients. In glioma patients, there was an increased risk of death in Black patients compared to White patients as well as for those living in areas of highest poverty. Ependymoma in less educated areas and areas with greater language isolation showed significantly increased risk of death compared with more educated and less language isolated areas.

### Multivariable analysis

We then undertook multivariable analysis to determine which factors retained an effect on survival after controlling for mediating factors (Table 2). We started with a base model in which we controlled for demographic and tumor characteristics, then added treatment, and finally socioeconomic characteristics. In the base model, Black patients had worse survival compared to White patients (HR: 1.39, 95% CI: 1.14–1.70, p-value = 0.0014), and Hispanic patients had worse survival compared to non-Hispanic patients (HR: 1.36, 95% CI: 1.16–1.60, p-value = 0.0002). Other race showed no significant difference in survival compared with White race. After controlling for both treatment and socioeconomic characteristics, the HRs for both Black and Hispanic patients decreased, but remained significant (HR: 1.29, 95% CI: 1.04, 1.59, p-value = 0.0206; HR 1.29, 95% CI: 1.08, 1.53, p-value = 0.0051, respectively). Accounting for demographics, tumor type, and treatment modalities, patients in the highest quartile for county-level percent below poverty had poorer survival (HR: 1.27, 95% CI 1.03–1.57, p-value = 0.0282).

Overall, other tumor types had improved survival compared to gliomas, metastatic patients had poorer survival, patients receiving surgery had improved survival, and patients receiving radiation had improved survival initially, but survival worsened compared to those without radiation as follow-up time progressed (Supplemental Fig. 1).

When stratifying by tumor type and controlling for demographic, treatment, and socioeconomic characteristics (Supplemental Table 1), Black children diagnosed with ependymoma had a higher risk of mortality compared with White children, while the increased mortality risk for Hispanic children did not quite meet statistical significance. For medulloblastoma patients, increased poverty worsened survival, but a lower level of education was associated with improved survival. Glioma patients receiving surgery had a decreased HR compared with those not receiving surgery.

### Extent of disease analysis

To determine whether survival disparities could be explained by a greater extent of disease at diagnosis, we examined the relationship of race and ethnicity with metastatic disease in a multivariable analysis, using models with and without consideration for socioeconomic characteristics (Table 3). In the base model, Hispanic patients had higher odds of being diagnosed with metastatic disease, but after controlling for socioeconomic characteristics, this difference was no longer significant. These results matched those found specifically in ependymoma, while in PNET/Pineal/ATRT tumors, the relationship remained significant even after controlling for socioeconomic characteristics. Black patients did not have an increased odds of metastatic disease overall or within any of the tumor types.

**Table 3:** Extent of disease at diagnosis, multivariable analysis, total.

Variable	Total N	Percent Reg/Dist	Controlled for: Demographic and Tumor Characteristics			Controlled for: Demographic, Tumor, and SES Characteristics		
			OR	OR 95% CI	p-value	OR	OR 95% CI	p-value
Year of Diagnosis (continuous)			0.97	(0.95, 0.99)	**0.0145**	0.97	(0.95, 0.99)	**0.0148**
Female (ref) Male	808 988	27.35% 24.09%	0.84	(0.67, 1.04)	0.1129	0.84	(0.67, 1.04)	0.1116
White (ref) Black Other	1409 231 147	26.12% 23.38% 23.81%	0.89 0.94	(0.63, 1.25) (0.62, 1.42)	0.5097 0.7645	0.93 0.91	(0.65, 1.31) (0.59, 1.38)	0.6653 0.6520
Non-Hispanic (ref) Hispanic	1294 502	23.88% 29.88%	1.32	(1.03, 1.68)	**0.0272**	1.25	(0.96, 1.63)	0.0905
00 years 01–04 years (ref) 05–09 years 10–14 years 15–19 years	104 477 496 353 366	36.54% 29.98% 27.82% 21.25% 17.76%	1.28 0.88 0.63 0.49	(0.81, 2.04) (0.66, 1.17) (0.45, 0.88) (0.35, 0.70)	0.2945 0.3810 **0.0069** **<0.0001**	1.28 0.88 0.63 0.49	(0.80, 2.03) (0.66, 1.18) (0.45, 0.89) (0.34, 0.70)	0.2995 0.3952 **0.0078** **<0.0001**
Gliomas (ref) Ependymoma Medulloblastoma PNET/Pineal/ATRT Unspecified/other	771 409 387 153 76	24.51% 22.00% 29.20% 35.29% 17.11%	0.71 1.17 1.24 0.56	(0.52, 0.96) (0.88, 1.57) (0.83, 1.84) (0.30, 1.06)	**0.0252** 0.2803 0.2892 0.0745	0.71 1.18 1.24 0.57	(0.52, 0.96) (0.88, 1.58) (0.83, 1.83) (0.30, 1.07)	**0.0265** 0.2694 0.2913 0.0815
Less than 6 cm (ref) 6 cm or greater Missing	1029 250 517	23.62% 30.00% 27.27%	1.50 1.17	(1.09, 2.07) (0.90, 1.50)	**0.0132** 0.2386	1.50 1.17	(1.09, 2.07) (0.91, 1.51)	**0.0139** 0.2248
All Other HS or less (ref) Highest Quartile % HS or less	1286 510	24.57% 28.04%				1.16	(0.83, 1.63)	0.3809
All Other Below Poverty Level (ref) Highest Quartile % Below Poverty Level	1261 535	25.46% 25.79%				0.85	(0.61, 1.19)	0.3413
All Other Language Isolation (ref) Highest Quartile % Language Isolation	1313 483	24.83% 27.54%				1.12	(0.84, 1.49)	0.4479

CI = confidence interval; Dist=distant; HS = high school; N = number of individuals in each category; OR = odds ratio; Reg=regional.

Notes: Highest Quartile % of county-level factors indicates the greatest level of disadvantage. Significant values (p < 0.05) have been marked with bold.

### Stratified analysis by extent of disease

Next, to determine potential influences of our variables of interest on patient outcomes after diagnosis and during the treatment process, we performed a multivariable analysis stratified by extent of disease (Table 4). We used the same three survival models to examine mediation: a base model with demographic and tumor characteristics, one controlling for treatment, and finally controlling for treatment and socioeconomic factors. In the base model for localized disease, both Black patients and Hispanic patients had poorer survival (HR 1.35, p-value = 0.0131; HR 1.36, p-value = 0.0023). After controlling for treatment and socioeconomic factors, the HRs for both Black and Hispanic patients decreased, with the HR for Black patients no longer significant (HR: 1.24, p-value = 0.0954) while the HR for Hispanic patients remained significant (HR: 1.27, p-value=0.0304). A similar pattern for Black patients was seen in the models limited to metastatic disease. In the base model, Black patients had significantly poorer survival (HR: 1.53, p-value = 0.0323), and in the full model, the HR decreased and was no longer significant (HR: 1.41, p-value = 0.1026). Hispanic patients had significantly poorer survival even in the full model (HR: 1.38, p-value = 0.0462). Areas with high poverty were associated with decreased survival (HR:1.77, p-value = 0.0084), and higher language isolation was initially associated with better survival, but survival worsened in comparison to persons living in areas with lower language isolation as follow-up increased.

**Table 4:** Overall survival, multivariable analysis, by extent of disease.

Localized Disease at Diagnosis									
Variable	Controlled for: Demographic and Tumor Characteristics (N = 1330)			Controlled for: Demographic, Tumor, and Treatment Characteristics (N = 1315)			Controlled for: Demographic, Tumor, Treatment, and SES Characteristics (N = 1315)		
	HR	HR 95% CI	p-value	HR	HR 95% CI	p-value	HR	HR 95% CI	p-value
Year of Diagnosis	1.00	(0.98, 1.02)	0.7188	0.99	(0.97, 1.01)	0.3675	0.99	(0.97, 1.01)	0.3839
Male vs. Female (ref)	1.12	(0.94, 1.33)	0.2133	1.09	(0.92, 1.30)	0.3305	1.09	(0.91, 1.29)	0.3554
Black vs. White Race (ref) Other vs. White Race (ref)	1.35 1.14	(1.07, 1.72) (0.82, 1.57)	**0.0131** 0.4421	1.26 1.16	(0.99, 1.61) (0.84, 1.61)	0.0616 0.3745	1.24 1.11	(0.96, 1.59) (0.80, 1.55)	0.0954 0.5292
Hispanic vs. Non-Hispanic Ethnicity (ref)	1.36	(1.12, 1.66)	**0.0023**	1.32	(1.08, 1.62)	**0.0064**	1.27	(1.02, 1.57)	**0.0304**
Age 00 vs. Age 01–04 (ref) Age 05–09 vs. Age 01–04 (ref) Age 10–14 vs. Age 01–04 (ref) Age 15–19 vs. Age 01–04 (ref)	2.11 0.97 0.70 0.32	(1.40, 3.16) (0.74, 1.26) (0.51, 0.96) (0.21, 0.49)	**0.0003** 0.8171 **0.0294** **<0.0001**	2.21 0.93 0.71 0.36	(1.45, 3.36) (0.71, 1.22) (0.51, 0.99) (0.23, 0.56)	**0.0002** 0.5801 **0.0415** **<0.0001**	2.31 0.94 0.71 0.36	(1.51, 3.53) (0.71, 1.23) (0.51, 0.99) (0.23, 0.56)	**0.0001** 0.6393 **0.0451** **<0.0001**
Ependymoma vs. Glioma (ref) Medulloblastoma vs. Glioma (ref) PNET/Pineal/ATRT vs. Glioma (ref) Unspecified/Other site vs. Glioma (ref)	0.22 0.35 0.74 0.56	(0.17, 0.30) (0.25, 0.48) (0.53, 1.03) (0.27, 1.15)	**<0.0001** **<0.0001** 0.0736 0.1140	0.23 0.39 0.78 0.76	(0.17, 0.31) (0.27, 0.55) (0.56, 1.09) (0.37, 1.55)	**<0.0001** **<0.0001** 0.1469 0.4513	0.22 0.39 0.77 0.76	(0.16, 0.30) (0.27, 0.56) (0.55, 1.08) (0.37, 1.54)	**<0.0001** **<0.0001** 0.1264 0.4438
6 cm or greater vs. <6 cm (ref) Missing tumor size vs. <6 cm (ref)	1.13 1.06	(0.87, 1.46) (0.87, 1.29)	0.3560 0.5520	1.20 1.02	(0.92, 1.56) (0.84, 1.25)	0.1738 0.8198	1.21 1.03	(0.93, 1.58) (0.84, 1.25)	0.1601 0.7829
Radiation Tx v. No Radiation Tx (ref)				0.60	(0.42, 0.87)	**0.0061**	0.61	(0.42, 0.88)	**0.0076**
Surgery vs. No Surgery (ref)				0.34	(0.24, 0.49)	**<0.0001**	0.34	(0.24, 0.49)	**<0.0001**
Highest Quartile Percent Less than HS Ed vs. All Others (ref)							0.92	(0.70, 1.20)	0.5290
Highest Quartile Percent Below Poverty vs. All Others (ref)							1.16	(0.90, 1.49)	0.2516
Highest Quartile Percent Language Isolation vs. All Others (ref)							1.14	(0.90, 1.44)	0.2852
Time-dependent Age Time-dependent Site Time-dependent Radiation Time-dependent Surgery	1.02 0.99	(1.01, 1.02) (0.99, 1.00)	**<0.0001** 0.0529	1.01 0.99 1.06 1.03	(1.01, 1.02) (0.99, 1.00) (1.04, 1.08) (1.00, 1.05)	**<0.0001** **0.0131** **<0.0001** **0.0223**	1.01 0.99 1.06 1.03	(1.01, 1.02) (0.99, 1.00) (1.04, 1.08) (1.00, 1.05)	**<0.0001** **0.0119** **<0.0001** **0.0196**
**Regional/Distant Disease at Diagnosis**									
**Variable**	**Controlled for: Demographic and Tumor Characteristics (N** = **457)**			**Controlled for: Demographic, Tumor, and Treatment Characteristics (N = 454)**			**Controlled for: Demographic, Tumor, Treatment, and SES Characteristics (N = 454)**		
	**HR**	**HR 95% CI**	**p-value**	**HR**	**HR 95% CI**	**p-value**	**HR**	**HR 95% CI**	**p-value**
Year of Diagnosis	0.97	(0.94, 1.00)	**0.0346**	0.97	(0.94, 1.00)	**0.0285**	0.96	(0.93, 1.00)	**0.0265**
Male vs. Female (ref)	1.24	(0.96, 1.62)	0.0996	1.24	(0.95, 1.61)	0.1120	1.30	(1.00, 1.70)	**0.0495**
Black vs. White Race (ref) Other vs. White Race (ref)	1.53 1.04	(1.04, 2.25) (0.64, 1.70)	**0.0323** 0.8701	1.63 1.00	(1.10, 2.41) (0.60, 1.65)	**0.0144** 0.9970	1.41 1.00	(0.93, 2.14) (0.60, 1.68)	0.1026 0.9852
Hispanic vs. Non-Hispanic Ethnicity (ref)	1.35	(1.02, 1.80)	**0.0351**	1.36	(1.02, 1.81)	**0.0335**	1.38	(1.01, 1.88)	**0.0462**
Age 00 vs. Age 01–04 (ref) Age 05–09 vs. Age 01–04 (ref) Age 10–14 vs. Age 01–04 (ref) Age 15–19 vs. Age 01–04 (ref)	0.99 1.04 0.71 0.78	(0.58, 1.68) (0.74, 1.44) (0.47, 1.08) (0.51, 1.18)	0.9687 0.8361 0.1090 0.2402	1.18 1.01 0.77 0.76	(0.68, 2.04) (0.72, 1.42) (0.50, 1.19) (0.49, 1.17)	0.5502 0.9516 0.2370 0.2105	1.33 0.98 0.76 0.79	(0.76, 2.31) (0.70, 1.39) (0.49, 1.18) (0.51, 1.22)	0.3154 0.9265 0.2160 0.2839
Ependymoma vs. Glioma (ref) Medulloblastoma vs. Glioma (ref) PNET/Pineal/ATRT vs. Glioma (ref) Unspecified/Other site vs. Glioma (ref)	0.25 0.39 0.56 0.49	(0.16, 0.37) (0.27, 0.55) (0.37, 0.86) (0.21, 1.13)	**<0.0001** **<0.0001** **0.0084** 0.0925	0.28 0.44 0.63 0.58	(0.18, 0.43) (0.30, 0.65) (0.40, 0.98) (0.25, 1.36)	**<0.0001** **<0.0001** **0.0416** 0.2080	0.28 0.44 0.63 0.63	(0.18, 0.43) (0.30, 0.65) (0.40, 1.00) (0.27, 1.48)	**<0.0001** **<0.0001** **0.0494** 0.2864
6 cm or greater vs. <6 cm (ref) Missing tumor size vs. <6 cm (ref)	1.09 1.03	(0.74, 1.59) (0.77, 1.39)	0.6641 0.8416	1.19 1.00	(0.81, 1.75) (0.74, 1.35)	0.3791 0.9983	1.15 0.98	(0.78, 1.70) (0.72, 1.34)	0.4695 0.9002
Radiation Tx v. No Radiation Tx (ref)				0.46	(0.29, 0.74)	**0.0012**	0.48	(0.30, 0.77)	**0.0024**
Surgery vs. No Surgery (ref)				0.57	(0.41, 0.81)	**0.0014**	0.59	(0.42, 0.84)	**0.0028**
Highest Quartile Percent Less than HS Ed vs. All Others (ref) Highest Quartile Percent Below Poverty vs. All Others (ref) Highest Quartile Percent Language Isolation vs. All Others (ref)							0.76 1.77 0.57	(0.49, 1.18) (1.16, 2.71) (0.36, 0.92)	0.2246 **0.0084** **0.0214**
Time-dependent Radiation Time-dependent Highest Quartile Language Isolation				1.05	(1.02, 1.08)	**0.0003**	1.05 1.03	(1.02, 1.08) (1.01, 1.05)	**0.0003** **0.0128**

CI = confidence interval; HR = hazard ratio; HS = high school; N = number of individuals in each category.

Notes: Highest quartile % of county-level factors indicates the greatest level of disadvantage. Hazard ratio (HR) indicates the ratio of hazard of death by levels of covariates. Significant values (p < 0.05) have been marked with bold.

In survival analyses stratified by tumor type and extent of disease (Supplemental Table 3), Black patients had worse survival than White patients for metastatic ependymoma and medulloblastoma. PNET/Pineal/ATRT patients with metastatic disease and living in high poverty areas showed worse survival when compared with those living in lower-poverty areas. Patients with medulloblastoma living in areas of lower education had better survival than those living in higher educated areas.

### Treatment modality analysis

**Figure 1:**
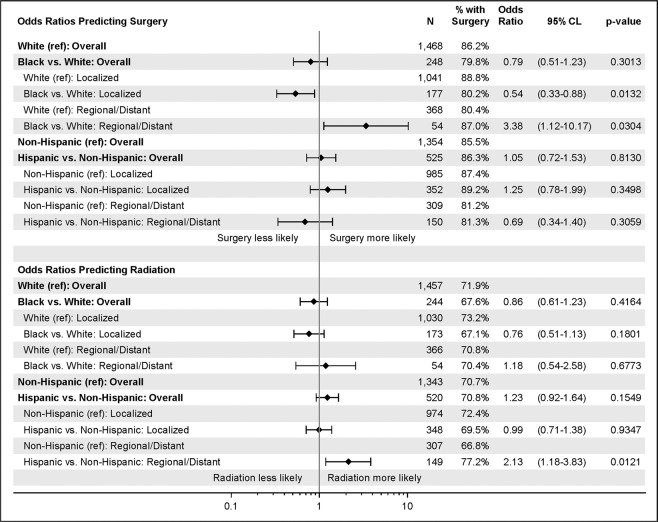
Comparing the receipt of surgery and radiation by race and ethnicity. Odds Ratio and 95% CL are demonstrated. Controlled for demographic, tumor, and SES characteristics.

Finally, we assessed the association of our variables of interest with the likelihood of patients being treated with surgery and/or radiation, the two treatment modalities measured in standard SEER data (Fig. 1). Overall, race or ethnicity were not predictive as to whether patients received surgery or radiation. When the analysis predicting surgery was stratified by extent of disease, Black patients with localized disease were less likely than White patients to undergo surgery (OR: 0.54, 95% CI: 0.33–0.88, p-value = 0.0132), and Black patients with metastatic disease were more likely than White patients to undergo surgery (OR: 3.38, 95% CI: 1.12–10.17, p-value = 0.0304). When the analysis predicting radiation was stratified by extent of disease, Hispanic patients with metastatic disease were more likely to receive radiation (OR: 2.13, 95% CI: 1.18–3.83, p-value = 0.0121).

## Discussion

In this study, we used SEER data to evaluate associations of demographic and socioeconomic variables with extent of disease at diagnosis and survival outcomes in childhood brain tumors. Black race and Hispanic ethnicity were both significantly associated with decreased overall survival after adjustment for mediating factors. Independently, those patients in areas of highest poverty had decreased overall survival when compared with areas of lower poverty. Our findings in pediatric brain tumors are consistent with previous studies showing disparities in outcomes for racial and ethnic minorities in other adult and pediatric cancers^3,6,7^. These prior studies have shown Black race and Hispanic ethnicity to be associated with poorer survival and/or later stage disease at presentation, although one study showed some mitigation by accounting for socioeconomic measures^7^.

Our findings on extent of disease at diagnosis suggest that Black race did not increase the chance of metastatic disease at presentation, and while Hispanic ethnicity did, it was in part explained by socioeconomic status. When we looked at survival stratified by extent of disease, we saw that Black and Hispanic patients with both metastatic and localized disease at diagnosis showed significant differences in survival likelihood compared to their White, non-Hispanic counterparts. However, once we controlled for treatment and socioeconomic factors, the survival difference was no longer significant for Black patients. Additionally, we saw evidence of differences in treatment by race and ethnicity when stratified by extent of disease, although this was somewhat limited by the lack of chemotherapy data. These data suggest that racial and ethnic disparities appear to be partially explained by post-diagnosis mediating factors that may fall in the pathway between race/ethnicity and poorer survival.

These post-diagnosis disparities depend in part on socioeconomic status, potentially implying lack of access to high quality care, leading to poorer overall outcomes, a root cause of disparities that has been described in other cancers^16^. Other factors on the patient/family side may also contribute to poorer outcomes post-diagnosis, such as language proficiency, stress about the cost of care, inability to take off time from work, and ability to secure transportation to treatment^17^. Socioeconomic factors also appeared to mediate the ethnic differences in the extent of disease at presentation overall. These findings overall are concordant with previous studies that have shown a correlation between disadvantaged socioeconomic status and survival outcome for both leukemias and solid tumors^2,9^. We had access to patients’ insurance status for only a subset of our population, and due to the smaller sample size, it was not included in multivariable analysis. As additional years of insurance data are collected, future studies may elucidate the association of insurance with extent of disease and survival. While potential biological differences in tumors between groups cannot be excluded and need to be further investigated, contributions to disparities based on socioeconomic status independent of race and ethnicity argue against an explanation based on racial/ethnic differences in tumor biology alone. To better understand underlying causes that contribute to the disparity of outcomes in pediatric brain tumors, patient-level data should be utilized in future studies to investigate both biological factors and pre/post-diagnosis treatment gaps in the care of children diagnosed with CNS tumors in the hopes of improving outcomes.

## Supplementary information

Supplementary information
