# Supplementary material for: Population-Based Analysis of Demographic and Socioeconomic Disparities in Pediatric CNS Cancer Survival in the United States
Source: Sci Rep. 2020 Mar 12;10:4588. doi: 10.1038/s41598-020-61237-2 (PMC7067886; doi:10.1038/s41598-020-61237-2)

Supplementary Information for

Population-Based Analysis of Demographic and Socioeconomic Disparities in Pediatric CNS Cancer Survival in the United States

Robert Fineberg,[1]* Shadi Zahedi,[1,2]* Megan Eguchi,[1,3] Muriel Hart,[1] Myles Cockburn,[1,3] Adam L. Green[1,2,3,4]

1 University of Colorado School of Medicine, Aurora, CO, USA

2 Morgan Adams Foundation Pediatric Brain Tumor Research Program, Aurora, CO, USA

3 University of Colorado Comprehensive Cancer Center, Aurora, CO, USA

4 Center for Cancer and Blood Disorders, Children's Hospital Colorado, Aurora, CO, USA

* These authors contributed equally.

Supplemental Table 1 Overall Survival, Univariate Analysis, By Tumor Category

Value	Ependymoma					Glioma					PNET/Pineal/ATRT					Medulloblastoma				
	N	Survival Estimate	HR	HR 95% CI	p-value	N	Survival Estimate	HR	HR 95% CI	p-value	N	Survival Estimate	HR	HR 95% CI	p-value	N	Survival Estimate	HR	HR 95% CI	p-value
Female (ref)	204	74.51%				359	34.34%				94	56.56%				160	67.59%			
Male	214	67.57%	1.34	(0.90, 1.99)	0.1458	429	30.88%	1.06	(0.88, 1.26)	0.5549	102	46.23%	1.34	(0.87, 2.07)	0.1839	233	62.85%	1.19	(0.82, 1.73)	0.3675
White (ref)	320	72.77%				607	33.37%				146	52.03%				328	64.38%			
Black	57	62.51%	1.55	(0.93, 2.60)	0.0955	115	25.38%	1.31	(1.03, 1.67)	**0.0263**	31	48.38%	1.21	(0.68, 2.17)	0.5148	35	67.09%	0.90	(0.47, 1.73)	0.7566
Other	40	68.61%	1.03	(0.52, 2.06)	0.9288	62	36.16%	0.98	(0.70, 1.38)	0.9211	17	45.61%	1.41	(0.68, 2.96)	0.3562	29	66.81%	0.92	(0.45, 1.88)	0.8099
Non-Hispanic (ref)	292	74.28%				576	34.02%				143	54.23%				283	67.65%			
Hispanic	126	63.05%	1.59	(1.06, 2.38)	**0.0253**	212	27.81%	1.20	(0.98, 1.47)	0.0706	53	40.95%	1.35	(0.84, 2.17)	0.2183	110	57.51%	1.51	(1.03, 2.22)	**0.0365**
00 years	28	66.73%	1.32	(0.62, 2.81)	0.4765	33	56.74%	0.95	(0.53, 1.71)	0.8600	26	25.18%	2.62	(1.48, 4.63)	**0.0010**	15	38.89%	1.72	(0.85, 3.51)	0.1328
01-04 years (ref)	166	71.09%				110	47.35%				77	51.18%				123	53.38%			
05-09 years	98	63.59%	1.26	(0.78, 2.04)	0.3500	217	20.74%	1.96	(1.44, 2.67)	**<.0001**	41	67.64%	0.49	(0.24, 0.98)	**0.0443**	142	66.14%	0.62	(0.41, 0.94)	**0.0258**
10-14 years	68	73.80%	0.86	(0.47, 1.58)	0.6288	186	23.69%	1.58	(1.15, 2.17)	**0.0046**	27	58.98%	0.68	(0.34, 1.37)	0.2828	70	80.83%	0.31	(0.16, 0.59)	**0.0004**
15-19 years	58	80.57%	0.68	(0.34, 1.36)	0.2722	242	40.25%	0.97	(0.71, 1.34)	0.8733	25	44.65%	0.83	(0.42, 1.64)	0.5950	43	77.09%	0.37	(0.17, 0.78)	**0.0087**
Localized (ref)	319	74.39%				582	35.62%				99	48.90%				274	72.05%			
Regional/Distant	90	59.79%	1.66	(1.08, 2.56)	**0.0207**	189	21.48%	1.62	(1.33, 1.99)	**<.0001**	54	39.40%	1.38	(0.87, 2.21)	0.1739	113	49.50%	2.11	(1.45, 3.05)	**<.0001**
Less than 6 cm (ref)	225	69.85%				406	34.91%				104	53.34%				298	67.18%			
6 cm or greater	85	71.58%	0.92	(0.55, 1.55)	0.7592	100	27.11%	1.18	(0.89, 1.55)	0.2457	32	41.90%	1.34	(0.76, 2.36)	0.3094	16	43.64%	1.47	(0.64, 3.37)	0.3666
No Radiation (ref)	116	70.49%				227	53.48%				72	37.58%				88	51.66%			
Radiation Administered	299	71.09%	0.86	(0.56, 1.31)	0.4842	554	23.54%	1.94	(1.55, 2.42)	**<.0001**	121	58.23%	0.42	(0.28, 0.65)	**<.0001**	301	69.79%	0.44	(0.30, 0.65)	**<.0001**
No Surgery (ref)	8	50.00%				222	16.97%				24	41.61%				7	28.57%			
Surgery	409	71.35%	0.38	(0.14, 1.03)	0.0578	566	38.48%	0.50	(0.41, 0.60)	**<.0001**	172	52.61%	0.58	(0.32, 1.05)	0.0714	385	65.66%	0.23	(0.09, 0.56)	**0.0012**
All Other HS or less (ref)	296	74.87%				552	33.99%				138	49.88%				296	64.57%			
Highest Quartile % HS or less	122	61.10%	1.56	(1.04, 2.34)	**0.0315**	236	28.95%	1.11	(0.92, 1.35)	0.2765	58	54.36%	0.84	(0.52, 1.37)	0.4839	97	65.53%	1.00	(0.65, 1.53)	0.9942
All Other Below Poverty Level (ref)	287	73.76%				540	35.04%				133	53.45%				298	66.77%			
Highest Quartile % Below Poverty Level	131	64.46%	1.44	(0.96, 2.15)	0.0813	248	26.77%	1.23	(1.02, 1.49)	**0.0285**	63	46.00%	1.14	(0.73, 1.80)	0.5670	95	58.72%	1.33	(0.90, 1.99)	0.1567
All Other Language Isolation (ref)	298	74.63%				572	32.99%				141	50.67%				302	66.63%			
Highest Quartile % Language Isolation	120	61.48%	1.52	(1.01, 2.28)	**0.0453**	216	31.09%	1.04	(0.85, 1.27)	0.7253	55	52.52%	0.89	(0.55, 1.45)	0.6528	91	58.19%	1.33	(0.88, 2.00)	0.1778
Private Insurance (ref)	143	82.05%				265	29.70%				69	52.55%				156	75.91%			
Public/No Insurance	88	63.39%	2.56	(1.37, 4.81)	**0.0033**	167	35.42%	0.89	(0.69, 1.16)	0.4062	35	56.23%	0.99	(0.49, 2.00)	0.9678	83	50.30%	1.90	(1.12, 3.21)	**0.0169**

CI= confidence interval;; HR=hazard ratio; HS= high school; N=number of individuals in each category.

Notes: Highest Quartile % of county-level factors indicates the lowest level of disadvantage. Hazard ratio (HR) indicates the ratio of hazard of death by levels of covariates.
Significant values (P<0.05) have been marked with bold.

Supplemental Table 2 Overall Survival, Multivariable Analysis, by Tumor Category

	Ependymoma (N = 405)			Glioma (N = 760)			PNET/Pineal/ATRT (N = 148)			Medulloblastoma (N =382)		
Variable	HR	HR 95% CI	p-value	HR	HR 95% CI	p-value	HR	HR 95% CI	p-value	HR	HR 95% CI	p-value
Year of Diagnosis	0.96	(0.91, 1.01)	0.1213	0.99	(0.97, 1.01)	0.2675	1.01	(0.95, 1.07)	0.7740	0.96	(0.91, 1.01)	0.1055
Male vs. Female (ref)	1.47	(0.97, 2.25)	0.0716	1.10	(0.91, 1.32)	0.3252	1.29	(0.78, 2.13)	0.3242	1.00	(0.67, 1.49)	0.9898
Black vs. White Race (ref)	2.05	(1.15, 3.65)	**0.0153**	1.21	(0.93, 1.58)	0.1633	1.38	(0.66, 2.91)	0.3948	0.76	(0.36, 1.58)	0.4621
Other vs. White Race (ref)	1.11	(0.52, 2.39)	0.7882	1.10	(0.77, 1.57)	0.6075	1.07	(0.45, 2.56)	0.8771	0.71	(0.32, 1.58)	0.4074
Hispanic vs. Non-Hispanic Ethnicity (ref)	1.62	(0.99, 2.66)	0.0550	1.18	(0.93, 1.48)	0.1715	1.51	(0.83, 2.74)	0.1729	1.23	(0.77, 1.97)	0.3949
Age 00 vs. Age 01-04 (ref)	1.05	(0.46, 2.38)	0.9023	2.00	(1.03, 3.87)	**0.0393**	3.76	(1.92, 7.37)	**0.0001**	2.09	(0.89, 4.90)	0.0917
Age 05-09 vs. Age 01-04 (ref)	1.22	(0.71, 2.10)	0.4679	1.18	(0.84, 1.65)	0.3422	0.34	(0.15, 0.76)	**0.0092**	0.80	(0.50, 1.27)	0.3453
Age 10-14 vs. Age 01-04 (ref)	1.10	(0.58, 2.09)	0.7804	0.75	(0.50, 1.12)	0.1605	0.26	(0.08, 0.88)	**0.0303**	0.42	(0.21, 0.84)	**0.0144**
Age 15-19 vs. Age 01-04 (ref)	0.63	(0.30, 1.31)	0.2156	0.31	(0.18, 0.53)	**<.0001**	0.14	(0.03, 0.67)	**0.0136**	0.38	(0.16, 0.90)	**0.0279**
6 cm or greater vs. < 6 cm (ref)	0.91	(0.53, 1.56)	0.7277	1.20	(0.90, 1.60)	0.2068	1.15	(0.62, 2.13)	0.6559	1.52	(0.63, 3.69)	0.3517
Missing tumor size vs. < 6 cm (ref)	0.78	(0.47, 1.28)	0.3221	1.03	(0.84, 1.27)	0.7484	0.95	(0.51, 1.77)	0.8802	1.11	(0.68, 1.82)	0.6674
Regional/Distant vs. Localized (ref)	1.69	(1.06, 2.67)	**0.0259**	1.37	(1.11, 1.69)	**0.0032**	1.59	(0.94, 2.67)	0.0828	1.80	(1.20, 2.70)	**0.0044**
Radiation Tx v. No Radiation Tx (ref)	0.25	(0.11, 0.61)	**0.0021**	0.91	(0.59, 1.40)	0.6713	0.58	(0.32, 1.06)	0.0782	0.30	(0.15, 0.62)	**0.0010**
Surgery vs. No Surgery (ref)	0.42	(0.12, 1.47)	0.1748	0.53	(0.43, 0.65)	**<.0001**	0.68	(0.30, 1.56)	0.3622	0.46	(0.15, 1.37)	0.1630
Highest Quartile Percent Less than HS Ed vs. All Others (ref)	1.11	(0.58, 2.10)	0.7599	1.05	(0.80, 1.39)	0.7115	0.54	(0.23, 1.24)	0.1442	0.43	(0.21, 0.89)	**0.0221**
Highest Quartile Percent Language Isolation vs. All Others (ref)	0.61	(0.25, 1.48)	0.2719	0.99	(0.77, 1.27)	0.9106	0.87	(0.48, 1.58)	0.6488	1.22	(0.74, 2.00)	0.4410
Highest Quartile Percent Below Poverty vs. All Others (ref)	1.11	(0.59, 2.09)	0.7505	1.24	(0.95, 1.61)	0.1144	1.69	(0.77, 3.68)	0.1905	1.99	(1.02, 3.87)	**0.0425**
Time-dependent Radiation	1.05	(1.02, 1.09)	**0.0018**	1.05	(1.02, 1.08)	**0.0002**				1.05	(1.02, 1.09)	**0.0035**
Time-dependent Highest Quartile Language Isolation	1.03	(1.00, 1.06)	**0.0313**									
Time-dependent Age				1.02	(1.01, 1.03)	**<.0001**	1.04	(1.02, 1.07)	**0.0001**			

CI=confidence level; HR=hazard ratio; HS= high school; N=number of individuals in each category.

Notes: Highest Quartile % of county-level factors indicates the lowest level of disadvantage. Hazard ratio (HR) indicates the ratio of hazard of death by levels of covariates. Significant values (P<0.05) have been marked with bold.

Supplemental Table 3 Overall Survival, Multivariable Analysis, by Tumor Category and Extent of Disease

	Ependymoma						Glioma					
	Localized (N = 315)			Regional/Distant (N = 90)			Localized (N = 572)			Regional/Distant (N = 188)		
Variable	HR	HR 95% CI	p-value	HR	HR 95% CI	p-value	HR	HR 95% CI	p-value	HR	HR 95% CI	p-value
Year of Diagnosis	0.932	(0.874, 0.994)	**0.0310**	1.012	(0.903, 1.134)	0.8334	0.990	(0.964, 1.016)	0.4397	0.957	(0.915, 1.001)	0.0543
Male vs. Female (ref)	1.466	(0.875, 2.455)	0.1465	1.661	(0.703, 3.927)	0.2474	1.015	(0.816, 1.263)	0.8946	1.368	(0.946, 1.978)	0.0958
Black vs. White Race (ref)	1.784	(0.927, 3.436)	0.0833	4.672	(1.105, 19.753)	**0.0361**	1.125	(0.823, 1.537)	0.4600	1.558	(0.901, 2.692)	0.1122
Other vs. White Race (ref)	0.947	(0.371, 2.415)	0.9093	1.018	(0.201, 5.166)	0.9826	1.331	(0.892, 1.986)	0.1612	0.623	(0.265, 1.464)	0.2776
Hispanic vs. Non-Hispanic Ethnicity (ref)	1.245	(0.680, 2.280)	0.4785	2.408	(0.850, 6.820)	0.0979	1.129	(0.856, 1.490)	0.3897	1.335	(0.852, 2.092)	0.2073
Age 00 vs. Age 01-04 (ref)	1.654	(0.540, 5.063)	0.3780	0.842	(0.237, 2.995)	0.7901	2.660	(1.264, 5.596)	**0.0099**	0.701	(0.152, 3.233)	0.6484
Age 05-09 vs. Age 01-04 (ref)	1.469	(0.782, 2.757)	0.2316	0.719	(0.193, 2.679)	0.6225	1.148	(0.768, 1.716)	0.5013	1.633	(0.890, 2.995)	0.1133
Age 10-14 vs. Age 01-04 (ref)	1.056	(0.480, 2.323)	0.8928	1.315	(0.367, 4.709)	0.6737	0.775	(0.485, 1.237)	0.2853	0.849	(0.428, 1.684)	0.6394
Age 15-19 vs. Age 01-04 (ref)	0.835	(0.365, 1.913)	0.6705	0.151	(0.016, 1.445)	0.1009	0.240	(0.126, 0.455)	**<.0001**	1.088	(0.561, 2.110)	0.8040
6 cm or greater vs. < 6 cm (ref)	1.000	(0.522, 1.913)	0.9991	0.467	(0.133, 1.636)	0.2341	1.169	(0.827, 1.653)	0.3765	0.935	(0.528, 1.657)	0.8176
Missing tumor size vs. < 6 cm (ref)	0.632	(0.335, 1.192)	0.1566	1.319	(0.515, 3.382)	0.5639	1.056	(0.831, 1.342)	0.6569	0.871	(0.581, 1.304)	0.5016
Regional/Distant vs. Localized (ref)												
Radiation Tx v. No Radiation Tx (ref)	0.321	(0.114, 0.898)	**0.0305**	0.187	(0.031, 1.110)	0.0650	1.003	(0.603, 1.666)	0.9921	0.570	(0.248, 1.309)	0.1851
Surgery vs. No Surgery (ref)	0.322	(0.069, 1.490)	0.1470	0.292	(0.029, 2.974)	0.2984	0.495	(0.385, 0.636)	**<.0001**	0.624	(0.420, 0.927)	**0.0195**
Highest Quartile Percent Less than HS Ed vs. All Others (ref)	0.991	(0.430, 2.282)	0.9822	1.834	(0.549, 6.125)	0.3240	1.021	(0.740, 1.410)	0.8992	1.310	(0.678, 2.532)	0.4211
Highest Quartile Percent Below Poverty vs. All Others (ref)	0.994	(0.460, 2.150)	0.9880	1.519	(0.424, 5.438)	0.5203	1.227	(0.904, 1.665)	0.1887	1.239	(0.681, 2.253)	0.4824
Highest Quartile Percent Language Isolation vs. All Others (ref)	1.246	(0.637, 2.436)	0.5208	0.170	(0.024, 1.184)	0.0735	1.032	(0.762, 1.396)	0.8406	0.801	(0.503, 1.277)	0.3516
Time-dependent Site												
Time-dependent Age							1.027	(1.016, 1.038)	**<.0001**			
Time-dependent Radiation	1.048	(1.007, 1.091)	**0.0212**	1.069	(1.002, 1.140)	**0.0428**	1.047	(1.019, 1.076)	**0.0010**	1.089	(1.024, 1.158)	**0.0069**
Time-dependent Surgery												
Time-dependent Highest Quartile Language Isolation				1.082	(1.021, 1.148)	**0.0080**						

Supplemental Table 3 (continued)

Variable	PNET/Pineal/ATRT Localized N = 97 HR	HR 95% CI	p-value	Regional/Distant (N = 51) HR	HR 95% CI	p-value	Medulloblastoma Localized (N = 270) HR	HR 95% CI	p-value	Regional/Distant (N = 112) HR	HR 95% CI	p-value
Year of Diagnosis	1.002	(0.924, 1.087)	0.9652	1.040	(0.924, 1.171)	0.5116	0.969	(0.907, 1.035)	0.3519	0.956	(0.876, 1.042)	0.3038
Male vs. Female (ref)	0.985	(0.505, 1.922)	0.9639	1.345	(0.462, 3.915)	0.5864	1.074	(0.608, 1.898)	0.8053	0.824	(0.427, 1.589)	0.5627
Black vs. White Race (ref)	2.078	(0.858, 5.033)	0.1049	1.903	(0.166, 21.854)	0.6054	0.465	(0.136, 1.583)	0.2203	0.797	(0.288, 2.205)	0.6619
Other vs. White Race (ref)	0.485	(0.105, 2.239)	0.3541	1.344	(0.396, 4.563)	0.6355	0.524	(0.164, 1.667)	0.2736	0.919	(0.274, 3.089)	0.8920
Hispanic vs. Non-Hispanic Ethnicity (ref)	1.949	(0.900, 4.221)	0.0905	1.163	(0.246, 5.500)	0.8494	1.514	(0.812, 2.823)	0.1914	0.835	(0.381, 1.832)	0.6531
Age 00 vs. Age 01-04 (ref)	6.588	(2.662, 16.308)	**<.0001**	1.892	(0.548, 6.537)	0.3133	1.333	(0.364, 4.881)	0.6646	3.809	(1.045, 13.877)	**0.0426**
Age 05-09 vs. Age 01-04 (ref)	0.314	(0.090, 1.092)	0.0686	0.324	(0.081, 1.298)	0.1115	0.759	(0.389, 1.480)	0.4183	0.800	(0.394, 1.624)	0.5372
Age 10-14 vs. Age 01-04 (ref)	0.154	(0.029, 0.817)	**0.0280**	0.868	(0.086, 8.788)	0.9047	0.463	(0.189, 1.130)	0.0908	0.369	(0.119, 1.145)	0.0844
Age 15-19 vs. Age 01-04 (ref)	0.106	(0.011, 0.987)	**0.0487**	0.033	(0.001, 1.067)	0.0544	0.589	(0.230, 1.504)	0.2683	0.000	(0.000, .)	0.9887
6 cm or greater vs. < 6 cm (ref)	0.664	(0.279, 1.581)	0.3552	1.113	(0.327, 3.788)	0.8643	2.054	(0.572, 7.383)	0.2700	1.240	(0.342, 4.495)	0.7434
Missing tumor size vs. < 6 cm (ref)	0.636	(0.281, 1.439)	0.2774	0.970	(0.273, 3.448)	0.9622	0.987	(0.504, 1.935)	0.9696	1.250	(0.503, 3.107)	0.6310
Regional/Distant vs. Localized (ref)												
Radiation Tx v. No Radiation Tx (ref)	0.603	(0.274, 1.327)	0.2083	0.432	(0.132, 1.413)	0.1649	0.158	(0.055, 0.453)	**0.0006**	0.967	(0.476, 1.967)	0.9271
Surgery vs. No Surgery (ref)	0.902	(0.317, 2.564)	0.8469	0.184	(0.028, 1.204)	0.0773	0.043	(0.009, 0.204)	**<.0001**	1.795	(0.226, 14.263)	0.5800
Highest Quartile Percent Less than HS Ed vs. All Others (ref)	0.803	(0.280, 2.304)	0.6833	0.141	(0.016, 1.250)	0.0785	0.390	(0.141, 1.078)	0.0695	0.323	(0.108, 0.965)	**0.0430**
Highest Quartile Percent Below Poverty vs. All Others (ref)	0.977	(0.359, 2.654)	0.9629	9.209	(1.121, 75.649)	**0.0388**	1.538	(0.613, 3.856)	0.3589	4.180	(1.404, 12.441)	**0.0102**
Highest Quartile Percent Language Isolation vs. All Others (ref)	1.429	(0.636, 3.211)	0.3878	0.569	(0.151, 2.141)	0.4046	1.681	(0.855, 3.305)	0.1321	0.601	(0.263, 1.375)	0.2280
Time-dependent Site												
Time-dependent Age	1.058	(1.022, 1.095)	**0.0012**	1.042	(1.006, 1.079)	**0.0220**						
Time-dependent Radiation							1.078	(1.019, 1.140)	**0.0092**			
Time-dependent Surgery												
Time-dependent Highest Quartile Language Isolation												

CI=confidence interval; HR=hazard ratio; HS= high school; N=number of individuals in each category.

Notes: Highest Quartile % of county-level factors indicates the lowest level of disadvantage. Hazard ratio (HR) indicates the ratio of hazard of death by levels of covariates.
Significant values (P<0.05) have been marked with bold.

Supplemental Fig. 1 Kaplan-Meier curves illustrating the overall survival for patients with and without radiation therapy.

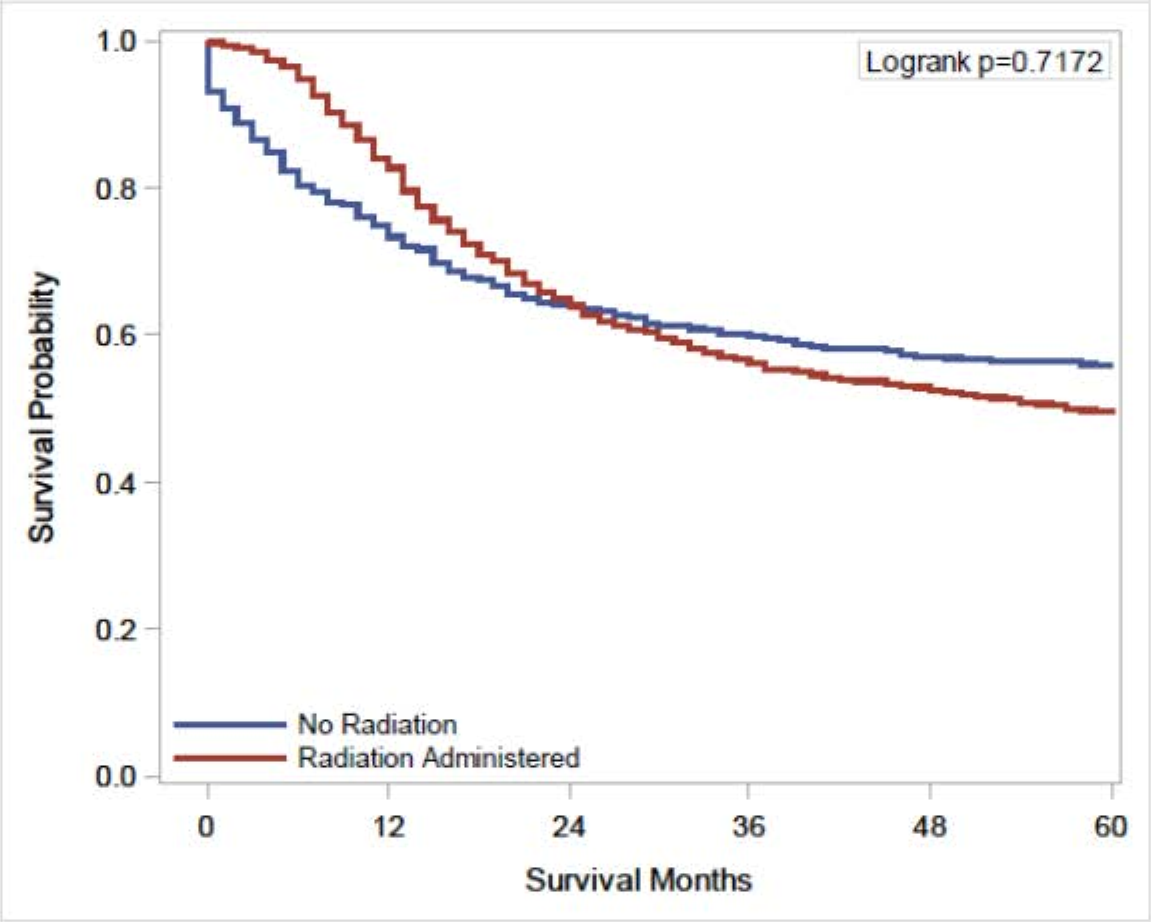

Supplement: Supplementary file 1 — Supplementary information [file 41598_2020_61237_MOESM1_ESM.pdf]
